# Impacts of Deciduous Leaf Litter and Snow Presence on Nymphal *Ixodes scapularis* (Acari: Ixodidae) Overwintering Survival in Coastal New England, USA

**DOI:** 10.3390/insects10080227

**Published:** 2019-07-30

**Authors:** Megan A. Linske, Kirby C. Stafford, Scott C. Williams, Charles B. Lubelczyk, Margret Welch, Elizabeth F. Henderson

**Affiliations:** 1 Center for Vector Biology and Zoonotic Diseases, The Connecticut Agricultural Experiment Station, P. O. Box 1106, New Haven, CT 06504, USA; 2 Vector-Borne Disease Laboratory, Maine Medical Center Research Institute, 81 Research Drive, Scarborough, ME 04074, USA

**Keywords:** integrated tick management, *Ixodes scapularis*, overwintering survival

## Abstract

Blacklegged ticks (*Ixodes scapularis* Say) are the vector for pathogens that cause more cases of human disease than any other arthropod. Lyme disease is the most common, caused by the bacterial spirochete *Borrelia burgdorferi* (Johnson, Schmid, Hyde, Steigerwalt, and Brenner) in the northeastern United States. Further knowledge of seasonal effects on survival is important for management and modeling of both blacklegged ticks and tick-borne diseases. The focus of our study was on the impact of environmental factors on overwintering success of nymphal blacklegged ticks. In a three-year field study conducted in Connecticut and Maine, we determined that ground-level conditions play an important role in unfed nymphal overwintering survival. Ticks in plots where leaf litter and snow accumulation were unmanipulated had significantly greater survival compared to those where leaf litter was removed (*p* = 0.045) and where both leaf litter and snow were removed (*p* = 0.008). Additionally, we determined that the key overwintering predictors for nymphal blacklegged tick survival were the mean and mean minimum temperatures within a year. The findings of this research can be utilized in both small- and large-scale management of blacklegged ticks to potentially reduce the risk and occurrence of tick-borne diseases.

## 1. Introduction

Tick-borne illnesses are a mounting public health concern in the United States, particularly in the Northeast. Pathogen transmission by blacklegged ticks (*Ixodes scapularis* Say) causes more human disease cases annually in the U.S. than any other arthropod vector [1]. Lyme disease, babesiosis, human granulocytic anaplasmosis, Powassan encephalitis, and relapsing fever can all be contracted from pathogens transmitted by blacklegged ticks [2,3,4]. Lyme disease occurrences have increased substantially over the past several decades, comprising an estimated 300,000 cases annually, and constituting over 70% of the reported cases of tick-borne disease in the contiguous U.S. [1,5,6]. While the documented range of blacklegged ticks spans portions of the Southeast and Midwest, it encompasses the Northeast in its entirety. In 2017, over 70% of reported confirmed or probable Lyme disease cases occurred in nine Northeast states: Connecticut, Maine, Massachusetts, New Hampshire, New Jersey, New York, Pennsylvania, Rhode Island, and Vermont [7].

With a continually expanding range, researchers and managers alike are searching for trends and reliable predictors for annual fluctuations in blacklegged tick densities. Studies have been conducted on multiple extrinsic factors such as anthropogenic effects on vector and pathogen dispersion [8,9], as well as the direct role of host dynamics on blacklegged tick abundances. Small mammals, such as white-footed mice (*Peromyscus leucopus* Rafinesque) and eastern chipmunks (*Tamias striatus* L.) [10,11], large-bodied hosts, such as white-tailed deer (*Odocoileus virginianus* Zimmermann) [12,13,14,15,16], or a combination thereof [17,18,19,20,21], have all been extensively researched to determine influences on the rise and spread of ticks and tick-borne pathogens. Even semi-irregular events such as acorn masting have been noted to have strong impacts on tick populations [22,23]. While these biotic factors play an important role in tick ecology, the large-scale dynamics of abiotic winter conditions on blacklegged tick survival need to be quantified as well.

It has been speculated that the climatic conditions of the previous winter can play a direct and crucial role in the survival and abundance of the nymphal stage blacklegged tick cohort and associated pathogens in the subsequent questing season [24,25,26]. Laboratory testing indicated that exposure to extreme cold and low humidity resulted in increased blacklegged tick mortality [24,27,28]. Tick survival is contingent on a temperature range between −10 and 35 °C with increased mortality associated with temperatures at either extreme [29]. Furthermore, humidity and soil saturation play a role in nymphal tick overwintering survival, requiring a minimum of 80% relative humidity [26,29,30]. These earlier studies provided valuable insight into overwintering effects on tick survival, but further field-based research is required to determine real-world patterns and their applications for predictive and adaptive management.

Our study quantified winter seasonal effects on nymphal blacklegged tick survival over the course of three winters in two different Northeast states: Connecticut and Maine. The multi-year nature of our project in conjunction with multiple locations provided a more in-depth analysis of variable winter effects on tick survival. The objective of our study was to determine the significant abiotic predictor(s) of nymphal blacklegged tick overwintering survival under various combinations of natural insulation. Specifically, we wanted to determine whether the presence, absence, or combination of leaf litter and snow accumulation would affect survival and how that varied between the two northeastern states during the harshest winter months of December, January, and February. Furthermore, we intended to determine differences in survival between years and locations based on several climatic variables and how that variability may aid in predicting impacts of overwintering effects on tick populations in the Northeast. Our intent was to provide a synopsis of overwintering conditions for these two study sites under specific conditions that can be used to further future research efforts and for potential tick management applications.

## 2. Materials and Methods

### 2.1. Study Sites

The field experiment was conducted over three winters (December through February): 2015–2016, 2016–2017, and 2017–2018 (hereafter referred to as years 1, 2, and 3, respectively) at two study sites, one in Hamden, Connecticut and the other in Cape Elizabeth, Maine. Two studies sites were selected as replicates for the study design in order to include the variability and gradient of winter weather conditions associated within the Northeast. The Hamden site (41°24′ N, 72°54′ W) is located at the Connecticut Agricultural Experiment Station’s Lockwood Farm. Woodlands consist of mature, upland hardwood stands with an understory composed of patches of dense shrubs. Fine sandy loam soils constitute the dominant soil type at the site [31]. The Cape Elizabeth site (43°34′ N, 70°13′ W) is typical of the dominant forest community of many post-agricultural woodlands in southern Maine with second-growth deciduous and mixed forests and a dense shrub layer. Loamy soils constitute the dominant soil type at all sites [32]. At both sites, tree canopy cover was uniform and representative of a typical northeastern, woodland environment and characteristic of blacklegged tick habitat.

At both study sites in a linear orientation on 2 m spacing, 24 plastic, cylindrical 4 L pots were placed in the ground approximately 20 cm deep, and backfilled with the excavated soil to approximately 6 cm from the top. Pots remained in their dedicated subterranean locations for the entirety of the study. Three ~5 cm diameter holes were cut from the lid and bottom of each pot and covered in a fine mesh fabric to allow flow of both air and water. Each pot housed three cylindrical, ~5 cm long plastic vials each containing 15 unfed, lab-reared, and pathogen-free *I. scapularis* nymphs (*n* = 1080 nymphs/site). Holes were cut in either end of each vial and covered with a fine mesh to contain nymphs within. Our intent was to make climatic conditions within the confines of pots and vials comparable to natural conditions while containing overwintering nymphs such that they could be easily relocated in spring.

### 2.2. Treatment Assignments

We used a randomized block design to assign each of the 24 pots to one of four treatments (six replicates/treatment). Treatments consisted of leaf removal (LR), snow removal (SR), a combination of both leaf and snow removal (LRSR), and a control, in which there was no manipulation of natural insulating barriers. In all 24 pots, the three vials containing nymphs were placed directly on top of the soil along with a data logger (HOBO^®^ Pro v2 Temp/RH; Onset Computer Corp., Bourne, MA, USA) programmed to record hourly temperature and relative humidity. In the control treatment, leaf litter that had been removed was placed over the top of the vials and the data logger, the lid was replaced, and a few leaves were placed over the lid. A similar process was used for the SR treatment with the intent to confine the nymphs and duplicate the amount of leaf litter comparable to the surrounding area. Leaf litter was not replaced in the LR and LRSR treatments; vials and data loggers were placed on the soil and the lid was replaced. In addition, three data loggers were affixed to metal stakes 1.0 m above ground level within the pot array that recorded ambient temperature and relative humidity data.

Any snow that accumulated over the winters was removed from approximately 4.0 m^2^ around each pot in the SR and LRSR treatments, but was not removed from control or LR treatments. We covered pots in the SR only treatment with a mesh cloth to facilitate snow removal without disturbing leaf litter. Where appropriate, snow was removed immediately after each weather event that resulted in any accumulation exceeding approximately 1.0 cm, and LR and LRSR treatment pots were checked and cleared every few days for leaf litter. For Maine, snow accumulation was measured after snow events and recorded weekly in the absence of additional snowfall. In Connecticut, the Lockwood Farm weather station reported and recorded snow accumulation on a daily basis for each year. Tick vials and data loggers were deployed in early November and retrieved in mid-April, after snow melt. Percent nymphal survival for each vial in each pot for each year was determined and recorded thereafter.

### 2.3. Statistical Procedures

#### 2.3.1. Two-Way Analysis of Variance

We initially conducted a two-way analysis of variance (ANOVA) to determine if significant differences existed in nymphal survival between treatments. Because survival data were highly variable for each tube containing nymphs, we determined percent survival of all nymphs for each treatment, for each year, with the locations combined for this preliminary analysis. Percent survival was the subject, and year and treatment type were factors. The Tukey honest significant difference (HSD) test with an alpha value of ≤0.05 was used for multiple comparison tests between treatments.

#### 2.3.2. Multiple Linear Regression

We ran a multiple linear regression model to determine the significant predictor(s) of nymphal overwintering survival. A maximum model was created using treatment type, and within each treatment type, mean minimum temperature, maximum temperature, mean temperature, mean relative humidity, mean vapor pressure deficit, location and year were used as predictors. Treatment type and location were applied as nominal, dummy variables [33]. A forward stepwise regression procedure was conducted to determine the most significant predictors of a combination thereof using an alpha value of ≤0.05 for inclusion. Treatment type was retained within the stepwise selection procedure to determine overall effects of abiotic variables across treatment types on overwintering tick survival.

#### 2.3.3. One-Way Repeated Measures ANOVA

Based on the results of the forward stepwise selection procedure, we conducted a one-way repeated measures (RM) ANOVA on ranks on input variable(s) deemed to be significant predictor(s). The Tukey HSD test with an alpha value of ≤0.05 was used for multiple comparison tests across treatments within each year. SigmaPlot (Version 13, Systat Software, Inc., San Jose, CA, USA) statistical software was used for all statistical analyses.

## 3. Results

### 3.1. Two-Way Analysis of Variance

Combined percent survival data passed the Shapiro–Wilk normality test (*p* = 0.341) and Brown–Forsythe test for equal variance (*p* = 1.000). Significant differences did not exist in percent nymphal survival between the three winters (F_2,6_ = 1.758, *p* = 0.251). However, significant differences did exist between treatments (F_3,6_ = 9.494, *p* = 0.011). Tukey HSD detected significantly greater nymphal survival in the control treatment compared to both LRSR (*p* = 0.008) and LR (*p* = 0.045) treatments only (Table 1).

### 3.2. Multiple Linear Regression

With treatment type retained, initial observations of the maximum model indicated that the following variables were statistically significant predictors for mean nymphal blacklegged tick overwintering survival; year (*p* = 0.004), mean minimum temperature (*p* = 0.023), and mean temperature (*p* < 0.001). As a result, the final model produced by the forward stepwise regression retained the same variables with *p* values of <0.001, 0.025, and <0.001, respectively. Year coefficient resulted in a 0.117 (SE ± 0.028) unit increase in nymphal survival. Mean minimum temperature resulted in a 0.011 (SE ± 0.005) unit decrease in survival and mean temperature resulted in a 0.079 (SE ± 0.009) unit increase.

### 3.3. One-Way Repeated Measures ANOVA

**Figure 1:**
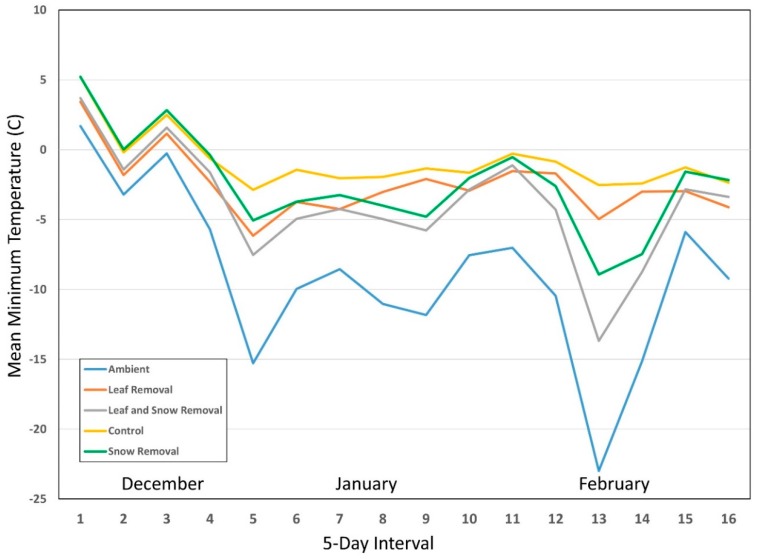
Year 1 mean minimum temperatures (°C) for all four treatment types and ambient temperature for 5-day intervals starting from mid-December through to the end of February for Connecticut and Maine combined.

**Figure 2:**
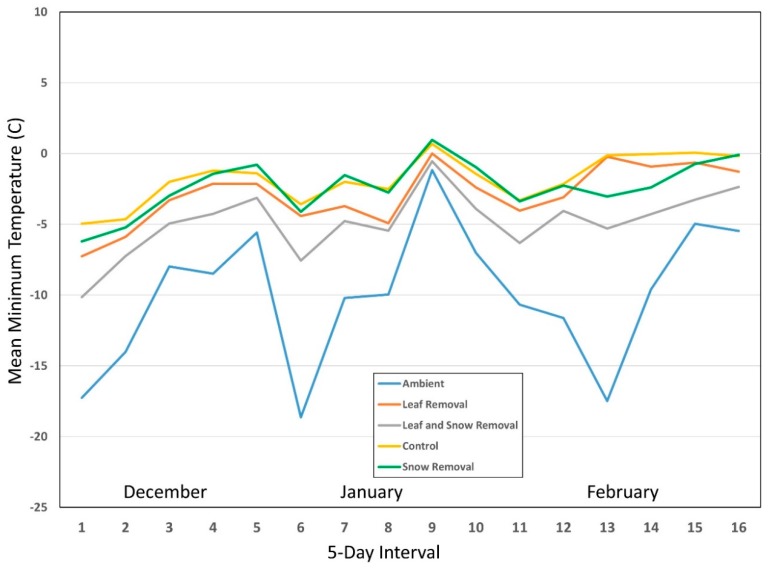
Year 2 mean minimum temperatures (°C) for all four treatment types and ambient temperature for 5-day intervals starting from mid-December through to the end of February for Connecticut and Maine combined.

**Figure 3:**
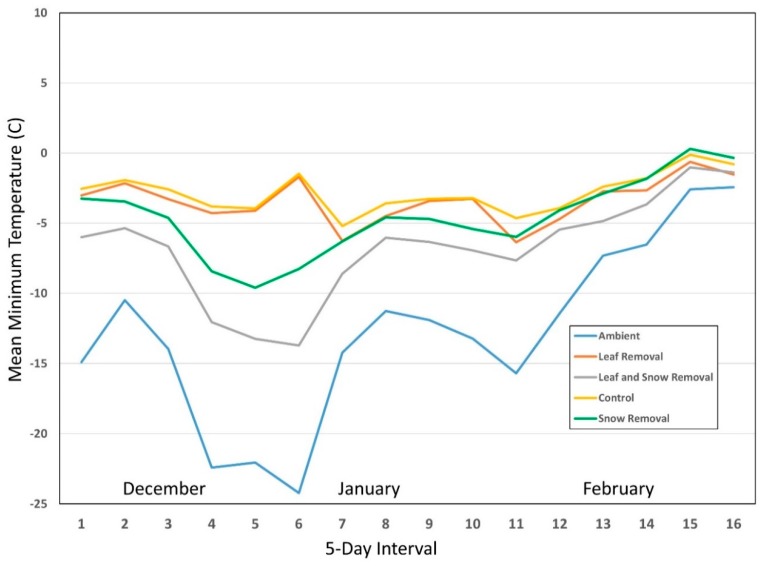
Year 3 mean minimum temperatures (°C) for all four treatment types and ambient temperature for 5-day intervals starting from mid-December through to the end of February for Connecticut and Maine combined.

Because of high variability within predictors, we determined mean temperature for the 12 pots within each treatment (ME = 6, CT = 6) for each year for 5-day intervals starting from mid-December through to the end of February (December 15–19, December 20–24, etc.). We used this time of year because it was when the snow and leaf removal treatments would have had the most combined influence on nymphal survival. While the leaf removal treatment would have had an effect on survival throughout all three winters, snow removal would only have a comparative effect during months when snow was likely to be present. We also determined mean minimum temperature for each of the 12 pots/treatments for the same 5-day intervals for each year (Figure 1, Figure 2 and Figure 3). We ran one-way RM ANOVA on ranks for both mean minimum (Table 2) and mean (Table 3) temperatures with 5-day interval as the subject, and treatments (control, SR, LR, LRSR, ambient) as factors in each of the three winters.

For mean minimum temperature ANOVA on ranks, the control was always significantly greater than both ambient and LRSR groups for all three years (Table 2). Snow removal and LR fluctuated from year to year between being statistically similar to both control and LRSR, but they were always significantly greater than ambient. Leaf removal/snow removal and ambient groups were statistically similar for all three years.

For mean temperature ANOVA on ranks, the control was always significantly greater than both the ambient and LRSR groups for all three years (Table 3). Snow removal and LR fluctuated from year to year between being statistically similar to the control, LRSR, and ambient groups. Leaf removal/snow removal and ambient groups were statistically similar for all three years as well. The lack of consistent statistical differences between SR and LRSR, and LR, appears to be due in part to the inconsistent insulating effect of snow accumulation on overwintering ticks. While leaf litter presence remained constant throughout the three winters, snow accumulation occurred sporadically with even more inconsistencies in its duration in the two coastal locations within the three-month period of December–February (Table 4).

## 4. Discussion

We found there were significant differences in nymphal blacklegged tick overwintering survival between treatments. For all three winters, regardless of location, the control pots had significantly greater survival of *I. scapularis* nymphs compared to LRSR and LR. These results indicate that leaf litter removal negatively impacted nymphal survival, which suggests that its presence provides a consistent insulative barrier from winter conditions. Although snow may act as additional insulation from the elements, its inconsistent and potentially short-term effects did not provide the same level of protection that leaf litter did in this study. Indeed, the impact of snow in future years may be less of a factor in overwintering survival as winters have warmed three times faster than summers in recent decades. Although warmer air can hold more moisture, winters are projected to be even milder in the century ahead with a trend of decreasing snowfall as indicated by a decreasing S/P ratio (ratio of snow to total precipitation) in the Northeast [34,35].

Based on previous studies, we were aware that both temperature and relative humidity are major factors in tick survival at any life stage. In particular, temperatures lower than −10 °C and relative humidity lower than 80% will result in significant mortality [26,29,30]. Based on the product of the stepwise selection procedure, the only significant predictors for survival in relation to our overwintering treatment types were year and mean minimum and mean temperature. We expected each year to have varying effects on survival as winter conditions can differ significantly annually. However, it was of interest that relative humidity and vapor pressure deficit were not significant predictors for tick survival. Our data loggers recorded that pots retained an average relative humidity of 93% throughout the three winters regardless of treatment type or location, which is notably greater than our ambient values due to the fact that relative humidity is highest closest to the ground [36]. Perhaps winter relative humidity levels do not play as crucial a role in tick survival compared to the summer questing season [37]. Conversely, there was variability in survival between treatment types corresponding to mean and mean minimum temperatures within each year.

Winters with consistently lower mean temperatures compared to other sampling years resulted in increased mortality. As mean temperature increased, so did survival (0.079 ± 0.009). Mean minimum temperature seemed to play a more complex role in overwintering survival between treatments. While the coefficient on its own caused a 0.011 unit decrease in survival when mean minimum temperature increased, based on observed trend lines in Figure 1, Figure 2 and Figure 3, the effects varied dramatically based on treatment type as well as what appears to be a threshold temperature. When mean minimum temperatures dropped below approximately −6.6 °C within pots, the trend lines separate between treatment types, and LRSR mean minimum temperatures plummeted following ambient temperatures. Temperatures in the SR and LR treatments also dropped significantly depending on year, most likely due to variations in snow accumulation during extreme cold events in addition to the presence or absence of leaf litter, respectively. This once again ties back into laboratory testing where temperatures lower than −10 °C resulted in increased mortality, but with the added complexity of field dynamics that either diminish or increase chances for survival. The consistent leaf litter in addition to the varying degrees of snow accumulation and duration resulted in greater overall survival with the control treatments compared to the other treatment types based on both mean minimum temperature and mean temperature over the course of each winter season. It should be noted that results from our study represent the maximum mortality of nymphs exposed to winter conditions as it has been reported that ticks infected with some pathogens may be better suited to survive winter temperatures [38] than the uninfected, lab-reared ticks that were included in this study.

These findings are significant in regards to management applications on both small and large scales. From the small-scale perspective, this field study determined that leaf litter plays a more significant role in overwintering nymph survival than previously reported. While snow accumulation also plays a part, its presence is erratic, difficult to manipulate, and too variable to monitor for management purposes. However, leaf litter can be addressed prior to the overwintering season to aid in the reduction of tick survival. Surrounding residential properties, questing nymphs have been found in greatest abundance within woodland ecotones, specifically transitional areas under brush or shady overstory, as well as areas adjacent to stone walls [39,40]. Stafford and Magnarelli [40] suggested that these areas be maintained by mowing vegetation, cutting back the immediate overstory, and removing brush and leaf litter. From a residential standpoint, the removal of leaf litter from these areas would have a twofold impact: the first being the immediate exposure of nymphal ticks during questing season as well as the reduction of an insulating barrier during winter months. The reduction of ticks through exposure to seasonal conditions in and surrounding residential properties would likewise reduce opportunities for nymphs to attach to human hosts and infect them with disease-causing pathogens [40,41].

From the large-scale perspective, we identified two key predictors for overwintering survival of nymphal stage blacklegged ticks: mean minimum and mean temperature. The significant predictors produced by our regression model can be used to develop more accurate estimates of tick population sizes to assimilate into integrated tick management (ITM) strategies and vector climate models. Climate warming may increase winter survival, shorten lifecycles, lengthen seasonal activity, and increase geographic expansion of ticks and their hosts [42]. For example, milder and shorter winters are favoring northward expansion of white-footed mice, the major reservoir host for several pathogens associated with *I. scapularis* [43].

ITM strategies can be highly effective when two control techniques are integrated, but the efficacy of those strategies is conditional on the abundance of the vector and the infection rate of pathogens [44,45,46,47,48]. Field studies such as ours bolster the accuracy and precision of this second point of ITM effectiveness by aiding in the prediction of annual fluctuations in vector abundance that can then be applied to management and control strategies. For instance, utilizing mean and mean minimum temperature predictors can assist in identifying low blacklegged tick population years. On those years, targeted ITM strategies utilizing control methods such as acaricide application and/or fipronil bait boxes, both aimed at early life stages such as nymphs, may have the most significant impact on further reducing blacklegged tick densities within a year. A significant reduction of one life stage within a year could have a cascading effect on later life stages and consequently the entire population of blacklegged ticks. This increased knowledge from our study will yield more accurate predictions annually on spring/summer questing nymphal blacklegged tick population sizes, and by extension, annual fluctuations in tick-borne disease risk. More accurate predictions will allow for more informed decision-making by land owners and managers about the use of ITM strategies for tick control.

## 5. Conclusions

The findings of our study indicate that winter weather conditions have significant effects on overwintering nymphal blacklegged tick populations, regardless of location. Based on our study, we can make two management recommendations on both small and large scales. Residential landscape modifications can effectively reduce tick overwintering survival by strategically removing the protective barrier caused by leaf litter presence. Additionally, the incorporation of key predictive variables into ITM strategies can aid in the development of more accurate management based on tick population estimates on a yearly basis. Ultimately, our findings should impact the available resources for scientists, public land managers, private landowners, and vector control specialists to make informed management decisions that accommodate the population dynamics of the primary vector for *Borrelia burgdorferi*.

## Figures and Tables

**Table 1:** Percent survival and number of surviving, unfed, nymphal *Ixodes scapularis* over three winters in Connecticut (CT) and Maine (ME). Total survival values with the same letter assignment were not significantly different. For each winter, *n* = 24 pots for both CT and ME.

Treatments	Y1		Y2		Y3		Total
	CT	ME	CT	ME	CT	ME	
Control	94% 249	21% 56	61% 166	53% 145	54% 142	74% 167	59% A 925
SR	86% 239	23% 62	63% 153	28% 72	47% 123	33% 98	46% AB 747
LR	86% 226	3% 7	53% 138	24% 63	44% 111	52% 139	44% B 684
LRSR	77% 207	3% 7	40% 106	17% 44	52% 132	31% 84	36% B 580

Y1: year 1; Y2: year 2; Y2: year 3; SR: snow removal; LR: leaf removal; LRSR: a combination of both leaf and snow removal.

**Table 2:** Median minimum temperature rank values (°C) for years 1–3 (Y1, Y2, Y3) for all four treatment types and ambient conditions. Values with different letter assignments within columns denotes significant differences using Tukey HSD with α ≤ 0.05.

Treatment	*N*	Y1	Y2	Y3
Control	12	−1.4 ^A^	−1.7 ^A^	−2.9 ^A^
SR	12	−2.4 ^AB^	−2.3 ^A^	−4.6 ^AB^
LR	12	−2.9 ^B^	−2.7 ^AB^	−3.3 ^A^
LRSR	12	−3.8 ^BC^	−4.5 ^BC^	−6.2 ^BC^
Ambient	6	−8.9 ^C^	−9.8 ^C^	−12.6 ^C^

**Table 3:** Median mean temperature rank values (°C) for years 1–3 (Y1, Y2, Y3) for all four treatment types and ambient conditions. Values with different letter assignments within columns denotes significant differences using Tukey HSD with α ≤ 0.05.

Treatment	*N*	Y1	Y2	Y3
Control	12	0.7 ^A^	0.4 ^A^	−2.1 ^A^
SR	12	1.0 ^AB^	0.9 ^A^	0.1 ^AB^
LR	12	1.0 ^AB^	0.8 ^AB^	−0.6 ^A^
LRSR	12	1.4 ^B^	1.1 ^B^	0.3 ^AB^
Ambient	6	1.2 ^B^	1.0 ^B^	−0.2 ^B^

**Table 4:** Total number of non-snow days compared to days with snow for both Connecticut and Maine over the course of three winter seasons. For days with snow, average monthly snow accumulation and total average snow accumulation were also reported.

Location	Year	Month	# of Non-Snow Days	# of Snow Days	Avg. Monthly Snow Accumulation (cm)
CT	1	Dec	16	0	0.0
CT	1	Jan	25	6	8.5
CT	1	Feb	14	15	12.9
CT	2	Dec	13	2	1.0
CT	2	Jan	26	5	9.2
CT	2	Feb	16	13	18.2
CT	3	Dec	11	4	2.0
CT	3	Jan	16	15	11.4
CT	3	Feb	25	4	7.2
**Total**			162	64	7.8
ME	1	Dec	16	0	0.0
ME	1	Jan	0	31	9.6
ME	1	Feb	3	26	10.9
ME	2	Dec	0	16	10.7
ME	2	Jan	6	25	26.4
ME	2	Feb	0	28	45.6
ME	3	Dec	0	16	20.2
ME	3	Jan	0	31	57.4
ME	3	Feb	0	28	16.1
**Total**			25	201	21.9

## References

[1] Rosenberg R., Lindsey N.P., Fischer M., Gregory C.J., Hinckley A.F., Mead P.S., Paz-Bailey G., Waterman S.H., Drexler N.A., Kersh G.J. (2018). Vital Signs: Trends in Reported Vectorborne Disease Cases United States and Territories, 2004–2016. Mmwr Morb. Mortal Wkly. Rep..

[2] Telford S.R., Armstrong P.M., Katavolos P., Foppa I., Wilson M.L., Spielman A. (1997). A new tick-borne encephalitis-like virus infecting New England deer ticks. Ixodes dammini. Emerg. Infect. Dis..

[3] Ebel G.D., Berenbaum M., Cardé R.T., Robinson G.E. (2010). Update on Powassan virus: emergence of a North American tick-borne Flavivirus. Annual Review of Entomology.

[4] Krause P.J., Narasimhan S., Wormser G.P., Rollend L., Fikrig E., Lepore T., Barbour A., Fish D. (2013). Human Borrelia miyamotoi infection in the United States. N. Eng. J. Med..

[5] Eisen R.J., Eisen L. (2018). The blacklegged tick, *Ixodes scapularis*: An increasing public health concern. Trends Parasitol..

[6] Adams D.A. (2016). Summary of notifiable infectious diseases and conditions—United States, 2014. Mmwr. Morb. Mortal Wkly. Rep.

[7] Lyme D. https://www.cdc.gov/lyme/index.html.

[8] Kilpatrick H.J., LaBonte A.M., Stafford K.C. (2014). The relationship between deer density, tick abundance, and human cases of Lyme disease in a residential community. J. Med. Entomol..

[9] Linske M.A., Williams S.C., Stafford K.C., Ortega I.M. (2018). *Ixodes scapularis* (Acari: Ixodidae) reservoir host diversity and abundance impacts on dilution of *Borrelia burgdorferi* (Spirochaetales: Spirochaetaceae) in residential and woodland habitats in Connecticut, United States. J. Med. Entomol..

[10] Ostfeld R.S., Miller M.C., Hazler K.R. (1996). Causes and consequences of tick (*Ixodes scapularis*) burdens on white-footed mice (*Peromyscus leucopus*). J. Mammal..

[11] Schmidt K.A., Ostfeld R.S., Schauber E.M. (1999). Infestation of Peromyscus leucopus and Tamius striatus by *Ixodes scapularis* (Acari: Ixodidae) in relation to the abundance of hosts and parasites. J. Med. Entomol..

[12] Wilson M.L., Levine J.F., Spielman A. (1984). Effect of deer reduction on abundance of the deer tick (*Ixodes dammini*). Yale J. Biol. Med..

[13] Wilson M.L., Adler G.H., Spielman A. (1985). Correlation between abundance of deer and that of the deer tick, *Ixodes dammini* (Acari: Ixodidae). Ann. Entomol. Soc. Am..

[14] Wilson M.L., Telford S.R., Piesman J., Spielman A. (1988). Reduced abundance of immature *Ixodes dammini* (Acari: Ixodidae) following elimination of deer. J. Med. Entomol..

[15] Stafford K.C., DeNicola A.J., Kilpatrick H.J. (2003). Reduced abundance of *Ixodes scapularis* (Acari: Ixodidae) and the tick parasitoid *Ixodiphagus hookeri* (Hymenoptera: Encyrtidae) with reduction of white-tailed deer. J. Med. Entomol..

[16] Rand P.W., Lubelczyk C., Holman M.S., Combe E.H., Smith R.P. (2004). Abundance of *Ixodes scapularis* (Acari: Ixodidae) after the complete removal of deer from an isolated island, endemic for Lyme disease. J. Med. Entomol..

[17] Daniels T.J., Fish D., Schwartz I. (1993). Reduced abundance of *Ixodes scapularis* (Acari: Ixodidae) and Lyme disease risk by deer exclusion. J. Med. Entomol..

[18] Daniels T.J., Fish D. (1995). Effect of deer exclusion on the abundance of immmature *Ixodes scapularis* (Acari: Ixodidae) parasitizing small and medium-sized mammals. J. Med. Entomol..

[19] LoGiudice K., Duerr S.T.K., Newhouse M.J., Schmidt K.A., Killilea M.E., Ostfeld R.S. (2008). Impact of host community composition on Lyme disease risk. Ecology.

[20] Williams S.C., Stafford K.C., Molaei G., Linske M.A. (2018). Integrated control of nymphal *Ixodes scapularis*: Effectiveness of white-tailed deer reduction, the entomopathogenic fungus *Metarhizium anisopliae*, and fipronil-based rodent bait boxes. Vector Borne Zoonotic Dis..

[21] Brunner J.L., Ostfeld R.S. (2008). Multiple causes of variable tick burdens on small-mammal hosts. Ecology.

[22] Ostfeld R.S., Schauber E.M., Canham C.D., Keesing F., Jones C.G., Wolff J.O. (2001). Effects of acorn production and mouse abundance on abundance and *Borrelia burgdorferi* infection prevalence of nymphal *Ixodes scapularis* ticks. Vector Borne Zoonotic Dis..

[23] Ostfeld R.S., Canham C.D., Oggenfuss K., Winchcombe R.J., Keesing F. (2006). Climate, deer, rodents, and acorns as determinants of variation in Lyme-disease risk. Plos Biol..

[24] Vandyk J.K., Bartholomew D.M., Rowley W.A., Platt K.B. (1996). Survival of *Ixodes scapularis* (Acari: Ixodidae) exposed to cold. J. Med. Entomol..

[25] Hayes L.E., Scott J.A., Stafford K.C. (2015). Influences of weather on *Ixodes scapularis* nymphal densities at long-term study sites in Connecticut. Ticks Tick-Borne Dis..

[26] Brunner J.L., Killilea M., Ostfeld R.S. (2014). Overwintering survival of nymphal *Ixodes scapularis* (Acari: Ixodidae) under natural conditions. J. Med. Entomol..

[27] Stafford K.C. (1994). Survival of immature *Ixodes scapularis* (Acari: Ixodidae) at different relative humidities. J. Med. Entomol..

[28] Burks C.S., Stewart R.L., Needham G.R., Lee R.E., Mitchell R., Horn D.J., Needham G.R., Welborne W.C. (1996). Cold hardiness in the ixodid ticks (Ixodidae). Acarology IX: Volume 1, Proceedings.

[29] Burgdorfer W. (1989). Vector/host relationship of the Lyme disease spirochete, *Borrelia burgdorferi*. Rhem. Dis. Clin. N Am..

[30] Oliver J.H. (1996). Lyme borreliosis in the southern United States: A review. J. Parasitol..

[31] NRCS Connecticut Soils. Natural Resources Conservation Service. https://www.nrcs.usda.gov/wps/portal/nrcs/main/ct/soils/.

[32] Service S.C. (1974). Soil Survey, Cumberland County, Maine.

[33] Kleinbaum D.G., Kupper L.L., Nizam A., Rosenberg E.S. (2013). Applied Regression Analysis and Other Multivariable Methods.

[34] Planet C.A.C., Wake C. Indicators of Climate Change in the Northeast 2005. http://www.cleanair-coolplanet.org/information/pdf/indicators.pdf.

[35] Thibeault J.M., Seth A. (2014). Changing climate extremes in the Northeast United States: Observations and projections from CMIP5. Clim. Chang..

[36] Ahrens C.D. (2012). Meteorology Today: An Introduction to Weather, Climate, and the Environment.

[37] Berger K.A., Ginsberg H.S., Gonzalez L., Mather T.N. (2014). Relative humidity and activity patterns of *Ixodes scapularis* (Acari: Ixodidae). J. Med. Entomol..

[38] Neelakanta G., Sultana H., Fish D., Anderson J.F., Fikrig E. (2010). Anaplasma phagocytophilum induces *Ixodes scapularis* ticks to express an antifreeze glycoprotein gene that enhances their survival in the cold. J. Clin. Investig..

[39] Carroll M.C., Ginsberg H.S., Hyland K.E., Hu R. (1992). Distributoin of *Ixodes dammini* (Acari: Ixodidae) in residential suburban landscape by area application of insecticides. J. Med. Entomol..

[40] Stafford K.C., Magnarelli L.A. (1993). Spatial and temporal patterns of *Ixodes scapularis* (Acari: Ixodidae) in southcentral Connecticut. J. Med. Entomol..

[41] Stafford K.C. (2007). Tick Management Handbook: An Integrated Guide for Homeowners, Pest Control Operators, and Public Health Officials For The Prevention of Tick-Associated Disease.

[42] Ogden N.H., Lindsay L.R. (2016). Effects of climate and climate change on vectors and vector-borne diseases: Ticks are different. Trends Parasitol..

[43] Roy-Dufresne E., Logan T., Simon J.A., Chmura G.L., Millien V. (2013). Poleward expansion of the white-footed mouse (*Peromyscus leucopus*) under climate change: Implications for the spread of Lyme disease. PLoS ONE.

[44] Pérez de León A.A., Teel P.D., Li A., Ponnusamy L., Roe R.M. (2014). Advancing integrated tick management to mitigate burden of tick-borne diseases. Outlooks Pest Manag..

[45] Ginsberg H.S., Stafford K.C., Goodman J.L., Dennis D.T., Sonenshine D.E. (2005). Management of ticks and tick-borne diseases. Tickborne Diseases of Humans.

[46] Stafford K.C., Ginsberg H.S. (1993). Forum: Management of Lyme Disease (Integrated Pest Management for *Ixodes scapularis*: Principals and Prospects). Ecology and Environmental Management of Lyme Disease.

[47] Ginsberg H.S. (2001). Integrated pest management and allocation of control efforts for vector-borne diseases. J. Vector Ecol..

[48] Ginsberg H.S. (1993). Transmission risk of Lyme disease and implications for tick management. Am. J. Epidemiol..

